# Laser-based molecular delivery and its applications in plant science

**DOI:** 10.1186/s13007-022-00908-9

**Published:** 2022-06-11

**Authors:** Dag Heinemann, Miroslav Zabic, Mitsuhiro Terakawa, Jens Boch

**Affiliations:** 1 Hannover Centre for Optical Technologies, Leibniz University Hannover, Nienburger Str. 17, 30167 Hannover, Germany; 2 Institute of Horticultural Production Systems, Leibniz University Hannover, Herrenhäuser Str. 2, 30419 Hannover, Germany; 3 Cluster of Excellence PhoenixD, Leibniz University Hannover, Welfengarten 1, 30167 Hannover, Germany; 4 Department of Electronics and Electrical Engineering, Keio University, 3-14-1 Hiyoshi, Kohoku-ku, Yokohama, 223-8522 Japan; 5 Institute of Plant Genetics, Leibniz University Hannover, Herrenhäuser Str. 2, 30419 Hannover, Germany

**Keywords:** Photoinjection, Optoporation, Laser transfection, Transformation, Laser-tissue interaction

## Abstract

Lasers enable modification of living and non-living matter with submicron precision in a contact-free manner which has raised the interest of researchers for decades. Accordingly, laser technologies have drawn interest across disciplines. They have been established as a valuable tool to permeabilize cellular membranes for molecular delivery in a process termed photoinjection. Laser-based molecular delivery was first reported in 1984, when normal kidney cells were successfully transfected with a frequency-multiplied Nd:YAG laser. Due to the rapid development of optical technologies, far more sophisticated laser platforms have become available. In particular, near infrared femtosecond (NIR fs) laser sources enable an increasing progress of laser-based molecular delivery procedures and opened up multiple variations and applications of this technique.

This review is intended to provide a plant science audience with the physical principles as well as the application potentials of laser-based molecular delivery. The historical origins and technical development of laser-based molecular delivery are summarized and the principle physical processes involved in these approaches and their implications for practical use are introduced. Successful cases of laser-based molecular delivery in plant science will be reviewed in detail, and the specific hurdles that plant materials pose will be discussed. Finally, we will give an outlook on current limitations and possible future applications of laser-based molecular delivery in the field of plant science.

## Introduction and theoretical background

### Historic view on laser-based molecular delivery

Since the laser became available to research in 1960, it was established as a precise, versatile and contact-free tool in many areas of the life sciences [1]. They were used as optical tweezers, for microdissection of DNA and filaments, to stimulate cell fusion, and to trigger laser-induced molecular transport [2–6]. The latter was first demonstrated in 1984, using an ultraviolet (UV) microbeam (λ = 355 nm, ${t}_{P}$ = 5 ns) to perforate the cell membrane of normal rat kidney cells to introduce an exogenous plasmid into the cells [7]. Few years later, a similar approach was presented for the manipulation of plant cells and chloroplasts [8, 9]. The outstanding feature of this method was the high precision of the processing, which enabled single cell selectivity and reduced detrimental side effects.

**Fig. 1:**
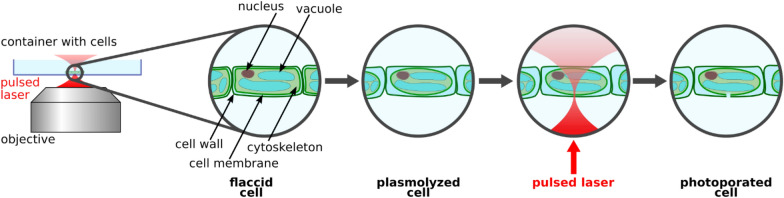
Sketch of a typical photoinjection experiment using an inverted microscopic setup and a pulsed laser source. The laser beam is focused onto the sample using an inverted microscope setup. A single laser pulse or a train of pulses facilitates of the cellular membrane and possibly the cell wall. The exact physical process of photoporation depends on the applied laser parameters and will be discussed in the following section. Plasmolyzing the plant cell prior to photoinjection supports the molecular uptake

As laser technology progressed, applications for laser-based molecular transport, here referred to as “photoinjection”, were increasingly developed. A sketch of a typical photoinjection experiment using a pulsed laser source is presented in Fig. 1. A major milestone was the introduction of near-infrared femtosecond pulsed (NIR fs) lasers and their application in transient transfection [6]. The great advantage of these systems is the reduced linear absorption of the NIR wavelengths in biological tissues. Thus, high penetration depths (up to 3 mm under specific conditions [10]) can be achieved and off-target effects outside the focal volume can be avoided, whereas shorter wavelengths e.g. in the visible range (VIS) had a residual risk of photoinduced changes even outside the focal volume [11]. For the processing of biological material at this wavelength, the simultaneous participation of several photons is required [12]. The photon densities required for this are achieved by the extremely high peak intensities confined in short time regimes, without any side effects occurring outside the focus.

Following up on this initial work, several studies investigated the fundamentals, possible optimizations, and various applications of photoinjection. For example, the high spatial selectivity of the approach was used to study local effects of translation of nuclear transcription factor E-26-like protein 1 (Elk-1) [13]. Elk-1 mRNA was selectively introduced into the dendrites or cell bodies of primary rat neurons by photoinjection. Translation of the mRNA in the dendrites resulted in cell death, whereas translation in the cell body did not. This allowed detection of localized effects on the translation that would have been difficult and invasive using other methods [13]. Another study used the approach to transfer the transcriptome of a rat astrocyte into a nondividing rat neuron which remodeled the cell type in 44% of the treated cells [14]. In vivo applications in full organisms have also been demonstrated by the transport of plasmids in zebrafish embryos, gene therapy in mice, or production of transgenic plants [10, 15, 16]. For a more detailed review of photoporation in mammalian systems we refer the reader to existing reviews on this matter [17, 18].

One of the greatest features of laser transfection is the single cell or even sub-cellular selectivity, but this severely limits the throughput for some applications. Therefore, different approaches have been presented to increase the throughput. Methods to produce multiple laser foci or to realize the positioning of the laser beam on the cell membrane in a (semi-)automated fashion increased the throughput, but only on a rather low level [19–23]. In contrast, approaches with more enhanced throughput rely on indirect laser-induced effects like pressure or shock waves (laser induced shock waves, LISW) generated by the absorbed laser energy or the application of nanomaterials [24–32]. The latter focuses the laser energy close to the cell membrane without the need to position the beam in a targeted manner [27–35]. The most widely used nanomaterials in this context are plasmonic gold nanoparticles, which combine several advantages for application in biological systems, such as high biocompatibility and easy conjugation [36]. Depending on the parameters, either the so-called nanoheater effect is used, in which the particles are abruptly heated by several 100 K via a short laser pulse and material in a small radius of a few 10 nm around the particle is thermally vaporized, or the enhancement of the laser field by near-field scattering is exploited (nanolens effect) [37, 38]. Both approaches have been successfully used to introduce various molecules into mammalian cells at high throughput (in the range of 1000 cells/second) [28, 32, 34]. For details on the underlying physics of laser-gold nanoparticle interaction we refer the interested reader to an extensive publication on this topic [39].

Taken together, laser-based molecular delivery has made great progress and yielded a variety of different distinct approaches, making it a promising tool for plant science. However, compared to the biomedical field, photoinjection has received only little attention in the plant community, so far, and the above described high throughput approaches have not been considered, yet. With this review, we aim to fill this gap by providing physical basics, an accurate picture of the available publications, challenges and future potentials of photoinjection in plant sciences.

The literature on laser-based molecular delivery faces the problem of incoherent terminology across the publications and a broad range of disciplines involved. Several terms have been used to describe laser-based molecular delivery under certain conditions, including laser microbeam cell surgery, laser micropuncture, laser transfection, photoporation, optoporation, and others [7, 16, 24, 40–46]. For consistency, we decided to use the term “photoporation” in the following whenever referring to the process of generating a transient laser induced opening of biological cells or organelles, and the aforementioned term “photoinjection” as the process of delivering a substance into the inner volume of a cell via photoporation. In accordance with existing terminology [17], the term photoinjection does neither imply a specific class of molecule or substance to be delivered nor a certain laser effect employed to achieve the delivery.

### Basic physics of photoinjection

**Fig. 2:**
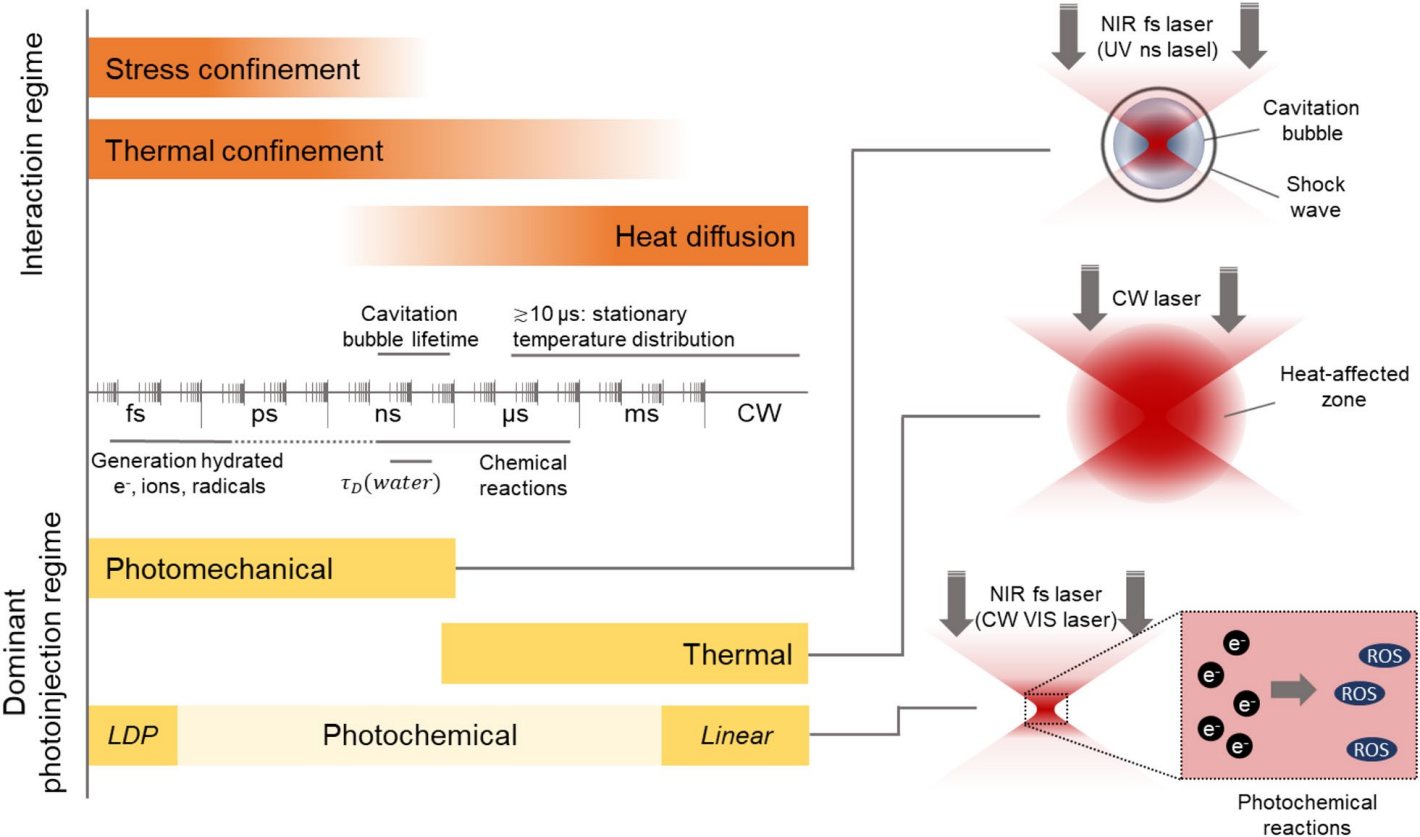
left: Overview comparing the time scales of different interaction and photopoinjection regimes. Note that the real values and borders vary largely on the respective conditions and can therefore only be regarded as rough estimates. Right: schematic depiction of the different interaction regimes. In the photomechanical regime, typically a single laser pulse with high energy (~ several 10 nJ for fs pulses) is applied, whereas the LDP requires multitudes of pulses with low energy (< 1 nJ) and high repetition rates (~ 80 MHz) to accumulate the photochemical effect. ${\tau }_{D}(water)$: thermal diffusion time in water for objective with high numerical aperture (NA), LDP: low-density plasma, NIR fs: near infrared femtosecond, CW: continious wave, ROS: reactive oxigen species

In the history of photoinjection, various laser sources with differing physical parameters have been applied. Some of these selections were based on the technical availability of specific laser sources to the respective point in time, but nowadays laser parameters can be selected to create specific interactions and should therefore be carefully considered when designing a respective experiment. Accordingly, we give a short overview of the different photoporation regimes and their basic physical concepts. Obviously, a large number of parameters impacts the laser-tissue interaction, including laser wavelength, applied intensity, pulse length, repetition rate, but also the scattering and absorption characteristic of the target material. In order to stick to an application-related explanation, we will mostly focus on the pulse length (or irradiation time) of the laser in respect to the timescales of relevant processes, as one major factor driving the nature of the laser interaction (see Fig. 2). Furthermore, most of the following considerations use water as a model because it represents the solvent of most biological processes, being present in excess compared to other molecules, and yielding good estimates of the tissue behavior [12, 47].

For photoinjection mechanisms, a basic distinction can be made between thermal, photomechanical and photochemical interactions (Fig. 2). A clear differentiation of the individual processes is hardly possible in practice and a change in irradiation can easily lead to a shift from one process to another. However, with the parameters used for photoinjection, one of the processes usually clearly dominates.

#### Photothermal regime

Thermal effects occur especially when using continuous wave (CW) lasers or laser sources with long pulse lengths (microseconds and longer). Under these conditions, the irradiation time is typically close to or above the thermal relaxation time, resulting in a steady state in which energy is supplied to the focal volume and simultaneously released to the environment in the form of heat [48]. To achieve efficient energy transfer from the laser to the medium, dyes (e.g., phenol red) are often used, which have an absorption range across the wavelength applied [44, 45, 49, 50]. The achieved local temperature gradients presumably cause denaturation of proteins in the immediate vicinity, an increase in the permeability of the cell membrane and, at sufficient temperatures, also evaporation of the medium with accompanying formation of long-lived (several seconds) gas bubbles [44, 45, 49]. The thermally induced change in cell permeability is presumably associated with a transition of lipids from the gel phase to a liquid crystalline phase [45, 51, 52]. Due to the relatively long time scales (well above stress confinement, where the laser pulse duration is shorter than the time required for the stress propagation out of the heated volume), thermoelastic effects are small in this regime and likely do not contribute to membrane permeabilization. Typical laser sources for this regime are argon ion lasers (CW, λ = 488 nm), diode lasers in the visible range or CW laser sources operating in the NIR wavelength range [44, 45, 49, 50, 53].

#### Photomechanical regime

For shorter pulsed irradiation in the range of nanoseconds or shorter, the energy input duration is shorter than the thermal relaxation time, so that thermal damage outside the focal volume can be neglected. As a rule of thumb, a limit of 1–10 µs is assumed for heat transfer in biological tissues [12, 54]. The material in the focal volume is disruptively ablated, i.e., direct material removal occurs followed by the formation of a cavitation bubble. Accordingly, a mechanical shock wave is generated, which propagates around the focal volume and can cause additional mechanical damage to biological structures [12, 55–57]. As the pulse length decreases, the pressure amplitude of the emitted pressure wave initially increases at constant applied energy, since the proportion of the pressure relieved by relaxation during the pulse length also decreases. However, this trend reaches a plateau as soon as the time range of the stress confinement is reached, which is typically in the picosecond range [12, 57]. Since the pulse duration is now shorter than the mechanical relaxation time, all of the mechanical energy is delivered in the shock wave. The amplitude is no longer dependent on the pulse length [12, 57]. At the same time, however, as the pulse length decreases, the required radiant exposure for optical breakdown decreases [12]. Accordingly, with shorter pulses (picoseconds, femtoseconds), the required energy input can be reduced, which in turn allows more controlled laser manipulation.

Historically, nanosecond pulsed UV lasers were applied with a wavelength around 340 nm, which is well above the absorption peaks of DNA and most proteins (reducing the risk of undesired photochemical damage), but still readily absorbed in aqueous media. More recent studies mainly relied on NIR fs lasers, typically operating at 800 nm, where titanium sapphire laser proved their power maximum. For the NIR fs lasers, amplifier systems yielding high intensity pulses (tens of nanojoules or more) at kHz repetition rates and oscillator systems with lower pulse energy (around 1 nJ) operating at MHz repetition rates can be separated [12, 58]. The former allows single pulse ablation following the aforementioned process, the latter can also operate in the low-density plasma regime (see below).

#### Photochemical and low-density plasma regime

With femtosecond laser systems, the interaction zone is limited to the volume where the intensities are higher than the thresholds of multi-photon absorption. Whereas the optical resolution is limited by diffraction as described by the Abbe criterion, the non-linear relation of multi-photon absorption on the laser intensity allows processing with resolution even below this limit. This also applies to photochemical processing in the so-called low-density plasma regime by ultra-short laser pulses [12]. Due to the short pulses, the electron density of the generated plasmas can be controlled very precisely. This is because in this time regime a significant number of electrons is directly excited by photoionization, in the range of optical wavelengths mainly by multiphoton absorption [12, 59]. This allows a deterministic relationship between irradiance and free electron density [12]. For longer pulses, the contribution from avalanche ionization is dominant, which leads to a rapid increase in electron density near the breakdown threshold that is difficult to control and is therefore associated with photodisruption [12]. The low-density plasma allows to provide a sufficient density of quasi-free electrons leading to photochemical effects, in particular chemical bond breaking via recombination without triggering photomechanical effects. The timescale from generation of quasi-free electrons, formation of ions and radicals (mainly derived from water molecules) and their recombination with biological molecules is in the range of nano- to microseconds and the effects accumulate over several laser pulses [12, 60–62]. Since the plasma volume can be smaller than the actual optical focal volume, extremely precise processing below the optical resolution limit can be achieved [12, 63]. Consequently, side effects should be lowest in this regime. At the same time, the small processing volume requires extremely high precision in sample positioning [64]. In addition, the high repetition rates of the laser systems used (typically 80 MHz) also result in a pulse-to-pulse distance that is below the thermal relaxation time in water for the focal volume (for example, with water as absorbing medium, NA = 1.3 and λ = 800 nm the resulting thermal diffusion time is ${\tau }_{D}$ $\approx$ 100 ns). Accordingly, thermal accumulation can occur over several pulses, so that for the thermal consideration not the individual pulse, but the total length of the applied pulse train must be considered [12].

Accordingly, from the physical-mechanistical point of view, processing in the low-density plasma regime would be the favorable approach, as it is associated with the highest precision and potentially the least side effects, suggesting minimal impairment of cell viability. However, in the application of photoporation, other factors such as practicability, efficiency of opening and also size of the pore enabled play essential roles. Therefore, it should be carefully considered to what extent the mentioned side effects have a significant impact on cell vitality and whether a larger processing volume in the application may even prove beneficial.

The former description presents a brief review of the different regimes. However, in context of this review we neglect several details on the laser-tissue interaction (thus, the areas of photoablation and photodisruption are not further differentiated and are combined under the term “photomechanical regime”) and the dynamics of resulting cavitation bubbles in the photomechanical regime for the sake of simplicity. We refer the interested reader to the extensive reviews on laser surgery [12, 56]. In summary, all three regimes discussed can be utilized for photoinjection. Approaches based on the photomechanical regime are the most widespread and usually associated with the highest injection efficiencies. Photochemical approaches, on the other hand, should at least theoretically have the highest precision and the least influence on the target cells.

## Applications of photoinjection in plant science

Within the biomedical field, a large variety of publications concerning different strategies and applications can be found. Contrary, the amount of publications describing photoinjection of plant cells is quite restricted – approx. 15 original publications since 1988 – although the potential applications appear manifold. Especially applications within plant breading, including genome editing techniques, rely on efficient tools for molecular delivery. An overview of the major publications, the applied laser parameters and the investigated plant species is given in Table 1. Hereafter, we give some general considerations on photoinjection in plants cells followed by a detailed discussion of the publications sorted by the utilized interaction regime.

**Table 1:** Overview of primary literature on photoinjection in plant cells (in chronological order)

Publication	Target species	Laser/mode of action	Remarks
Weber et al. [8]	*Brassica napus* L	λ = 343 nm, τ_P_ = 15 ns, E_P_ = several mJ, single pulse, mechanism: photoablation	First report on photoinjection in plant cells, photoinjection of stained plasmid DNA
Weber et al. [80]	*Brassica napus* L. cells and pollen grain	λ = 343 nm, τ_P_ = 17 ns, E_P_ = several mJ, single pulse, mechanism: photoablation	Photoinjection of stained plasmid DNA in pollen grains and cells, cell wall and membrane were opened by two consecutive laser pulses
Weber et al. [9]	*Brassica napus* L	λ = 343 nm, τ_P_ = 15 ns, E_P_ = several mJ, single pulse, mechanism: photoablation	Injection of bisbenzimide stained plasmid DNA in isolated chloroplasts, resealing of the membrane was estimated within 1.2 s after laser treatment
Guo et al. [69]	*Oryza sativa* L. cv. Japonica	λ = 355 nm, τ_P_ = 15 ns, E_P_ = 0.2–1 mJ, f_rep_ = 10 Hz, scanning irradiation mode, mechanism: photoablation	Transformation frequency of 4.8 * 10^–3^, regeneration of transgenic plantlet under kanamycin selection was demonstrated
Tirlapur & König [11]	*Arabidopsis thaliana* Columbia meristems	λ = 800 nm, τ_P_ = 180 fs, f_rep_ = 80 MHz, P = 9 mW, exposure time = 0.047 s, mechanism: photodisruption	First report of NIR fs laser photoinjection in plant cells, investigation of intercellular transport
Awazu et al. [81]	Tobacco BY-2	λ = 5.5, 5.75, and 6.1 µm, τ_P_ = approx. 10 ps, f_rep_ = bursts of 300 – 400 pulses at 100 Hz, exposure time = 100 s, optimal radiant exposure = 1.4 J/mm^2^ , mechanism: thermal	Wavelength corresponding to linear absorption peaks, transient expression of a reporter plasmid in max. 0.5% of the treated cells
Badr et al. [16]	Calli of *Triticum aestivum* L. cv. Giza 164	λ = 308 nm, τ_P_ = 6 ns, E_P_ = 2–4 mJ, f_rep_ = 200 Hz, scanning irradiation mode, mechanism: photoablation	Photoinjection of a 2.09 kb GUS vector, regeneration of 3 transgenic plants from 600 GUS positive calli under bialaphos selection
Schinkel et al. [64]	Tobacco BY-2	λ = 1064 nm, τ_P_ = 392 – 460 nJ, E_P_ = 17 ps, single pulse, mechanism: photodisruption	Only publication on the use of picosecond laser, efficiency for transient YFP expression approx. 2.5%
LeBlanc et al. [63]	*Arabidopsis* epidermal cells	λ = 750 nm, τ_P_ = 200 fs, f_rep_ = 80 MHz, P = 5 – 100 mW, exposure time = 0,64 µs, mechanism: photochemical (LDP)	Calculation on the low-density plasma regime, efficiency for 10 kDa FITC-dextrans = approx. 68%,
Mitchell et al. [70]	Tobacco BY-2 (mammalian CHO cells as reference)	λ = 800 nm, τ_P_ = 140 fs and sub 20 fs, f_rep_ = 80 MHz, exposure time = 40 ms, P = 70 mW (Gaussian beam) or 1.6 W (Bessel beam), mechanism: photodisruption	Comparison of different beam geometries (Gaussian beam with one or three foci, Bessel beam) and osmolar conditions, investigation of the dependence injection efficiency vs Stokes radius of the molecule
Maeno et al. [82]	*Euglena gracilis* (microalgae)	λ = 800 nm, τ_P_ = 100 fs, E_P_ = 80 nJ, f_rep_ = 1 kHz, scanning irradiation, mode (100 µm/s) mechanism: photodisruption	Delivery of a paramylon-binding aptamer-based fluorescent probe
Rukmana et al. [68]	Tobacco BY-2	λ = 800 nm, τ_P_ = 150 fs, E_P_ = 80 nJ, single pulse mechanism: photodisruption	Delivery of 20 kDa and 2 MDa FITC-dextrans, enzymatic pretreatment of the cell wall
Rukmana et al. [83]	Tobacco BY-2	λ = 800 nm, τ_P_ = 150 fs, E_P_ = 20 nJ, single pulse, mechanism: photodisruption	Delivery of polymeric particles (BODIPY, 80 nm)
Rukmana et al. [84]	Tobacco BY-2	λ = 800 nm, τ_P_ = 150 fs, E_P_ = 20 nJ, single pulse, mechanism: photodisruption	Delivery of particles of 4 different diameters (3.2, 26.7, 80, 110 nm), investigation of intracellular and intercellular particle diffusion

The stated mechanisms either state the reported terms or the most likely mechanism based on the given parameters. λ = wave length, τ_P_ = Pulse length, E_P_ = pulse energy, f_rep_ = pulse repetition rate, P = output power

### General considerations on the physical transport of molecules into plant cells

The majority of experiments employing photoporation has been conducted in mammalian systems. These provided the basis for photoporation in plant cells, however, several unique aspects related to the plant cell physiology need to be considered in order to establish a successful photoporation experiment. Here, we will briefly discuss some general considerations, before giving a detailed overview of published work in the following section.

Plant cells possess two main barriers, namely the cell wall and the cellular membrane, which naturally function to protect the cell from external components. Though the cell wall allows free diffusion of small molecules, the penetration of macromolecules and nanoparticles can be hindered. The exact cut-off values depend on a variety of parameters, including the plant species, the tissue, and the molecule (e.g. charge, 3D structure) under consideration as well as external factors like pH and temperature. For macromolecules, a size exclusion limit (SEL) in the range of 40 to 60 kDa has been established, whereas the SEL for particles (assumed as solid spheres), often considered in nanotoxicity, is reported between 5 and 20 nm [65–68]. Molecules below this range should be able to passively diffuse in the apoplastic space and might be delivered to the cell by single photoporation of the cell membrane. For molecules above the SEL, the cell wall needs to be actively permeabilized first. This can be facilitated by performing the photoinjection as a two-step mechanism, or punctation at the contact points between the membrane and wall in order to achieve permeabilization of both barriers with a single illumination [8]. In such intact plant cells, the inner pressure of the cell, the turgor, will provide a mechanical stability to the cell. This pressure will result in extrusion of cytoplasmatic content when the membrane is opened, and this outflow of cell content will hinder any inward transport [69, 70]. Accordingly, pre-treatment with a hyperosmotic buffer inducing plasmolysis of the cell is an efficient way to circumvent this. The photoporation-induced uptake of external liquid has been shown to lead to a restoration of the initial cell volume and to promote molecular uptake [8, 69, 70]. Furthermore, plasmolysis seems to result in a higher cell viability after photoporation. Hyperosmolar treatment can therefore be regarded as a central perquisite for photoporation of plant cells, although a well-adjusted balance between plasmolysis and toxicity due to the osmolar shock needs to be met.

Another approach is to enhance the permeability of the cell wall via enzymatic digestion up to the release of largely cell wall-free protoplasts. Protoplasts are sensitive to changes in osmolarity. Therefore, an active volume uptake as described in the previous paragraph cannot easily be established without impacting cell viability. Although protoplasts appear to be an attractive target to photoporation due to their lack of cell walls, reports on the photoporation of protoplasts have been of little success until now, which might be related to the lack of plasmolysis driven uptake and increased sensitivity to treatments [70].

In the usual experimental configuration, photoinjection allows permeabilization of the cellular membrane and thereby molecular transport into the cytoplasm. When substances are used that are destined for intracellular compartments (e.g., exogenous DNA targeted for the nucleus), the further transport needs to be considered. Possible spontaneous transport of plasmid DNA through nuclear pore complexes has been discussed in the past, but the amount of cytoplasmic DNA entering the nucleus on non-dividing cells is low (~ 0.1%) and its contribution to transfection is likely small, if relevant at all [71–74]. The size of the aqueous channel of nuclear pore complexes was estimated to be approx. 9 nm, allowing passive diffusion of molecules up to 40–60 kDa [75, 76]. Accordingly, larger molecules require active transportation or other means to overcome the nuclear membrane. Studies on photoinjection in mammalian cell cultures are therefore often based on temporary nuclear membrane degradation during mitotic cell division [17, 77]. This approach is feasible for proliferating cells, but significantly reduces the scope of application in non-dividing cells. The intuitive approach to consecutively puncture the cellular and the nuclear membrane via photoporation is theoretically possible but experimentally laborious and the probabilities of success have to be multiplied with each barrier resulting in a significant reduction of the expected efficiency [78]. Another possibility is provided by biochemical methods, such as nuclear localization sequences (NLS) in proteins that exploit active transport mechanisms into cellular compartments [79].

### Photoinjection of plant cells in the photothermal regime

Local temperature gradients induced by the absorption of laser light in the focal volume can be utilized to permealize the cellular membrane (see “Basic physics of photoinjection” section). To achieve this, the combination of CW laser radiation and absorbing dyes has been explored in mammalian cells as well as in plant cells [44, 45, 49, 50, 53]. Besides some success for the transient transfection of mammalian cells, only indirect evidence for permeabilization of the cells was given in plants [49]. Instead of an external absorbing dye, natural occurring absorption bands of cellular structures can be utilized [81]. The linear absorption by components of the cell wall and membrane could induce a perforation and allow molecular uptake without the need for focusing the laser. Bursts of picosecond laser pulses were used to minimize the thermal impact beyond the cell borders. With this approach, the small fluorescent dye DAPI was delivered to approx. 3% of treated BY-2 cells and a transient transfection efficiency of 0.5% was reached, based on a GFP marker vector [81]. As the approach doesn’t require focusing on the cell surface, the increased throughput is a clear advantage. However, absorption of laser energy by intracellular components might give rise to toxicity and the publication lacks information in this regard [81]. Taken together, because of the rather unspecific mode of action and the potential thermal impact, thermal approaches seem to be less promising.

### Nanosecond UV laser pulses for photomechanical photoinjection in plant cells

The use of UV laser microbeams pioneered the field of photoinjection in plant science [8, 9, 80, 85–88]. The applied nanosecond laser pulses together with the highly energetic photons primarily induce photomechanical effects to induce photoinjection (see Fig. 2). In early studies, delivery of DNA labeled with the fluorescent dye bisbenzimide into isolated chloroplasts of *Brassica napus* was reported using a laser (λ = 343 nm, ${t}_{P}$ = 15 ns) coupled to a fluorescence microscope [9]. Uptake of the labeled DNA was observed after applying a single laser pulse to the chloroplast membrane by fluorescence increase inside the organelle. Using video microscopy and extracellular added DNase, resealing of the introduced membrane opening was estimated within 1.2 s [9]. Delivery of a plasmid encoding a triazine resistance gene to chloroplasts inside an intact *B. napus* cell was tested by mechanical loading of the plasmid into a protoplast following photoporation of the chloroplast’s membrane. Although a transient expression of the transgene was observed in a small fraction of cells, no transgenetic plants could be regenerated from these traits [88]. As only a fraction of the protoplasts of a cell can be treated this way, it is possible that transgenic chloroplasts were not conserved during consecutive cell divisions. Using the same laser setup, intact cells of *B. napus* pretreated with hypertonic buffer were photoinjected with labeled DNA, as well. Noteworthy, two consecutive pulses were applied, the first perforating the cell wall and the second targeting the membrane. The plasmolyzed cells recaptured their original cell volume after laser application, implying a volume uptake through the laser-generated hole. Within this experiment, 80% of laser treated cell survived on a short term, while 50% kept their proliferation capacity [8, 80]. Further experiments proved the transient expression of an introduced transgene (a plasmid carrying bacterial glucuronidase (GUS) [89]) in 70% of the laser treated *B. napus* cells. 85 hygromycin resistant colonies were acquired out of 1,000 laser treated cells incubated with a plasmid encoding a hygromycin B resistance gene [8, 88, 90]. The authors stated the possibility to regenerate transgenic plants from this trait as well as the possibility to treat microspores from immature *B. napus* pollen grains, however no data was presented to support these statements [88]. These basic experiments marked the starting point of plant photoinjection.

Using comparable systems equipped with lasers in the UV range and with nanosecond pulse durations, other groups tried to establish transgenetic plants. Embryonic calli of *Oryza sativa* L. cv. Japonica were photoinjected with an UV microbeam in a semi-automated fashion. Using the GUS gene as marker, transient expression occurred in about 1 out of 50,000 cells and using kanamycin as selection marker, complete transgenic plantlets could be regenerated from photoinjected calli [69]. Transgenetic plants resistant to *Sclerotinia sclerotiorium* were produced by photoinjecting cotyledonary petiole cells of *B. napus*. Plants were grown to the T_2_ generation under kanamycin selection and the majority of the plants showed resistance to *S. sclerotiorium*, while incidence for stable integration of the transgenes was found in two plants of a smaller sample check of the T_0_ generation by southern blot analysis [91]. In *Triticum aestivum* L. cv. Giza 164, transformation with a marker gene for herbicide bialaphos resistance was investigated [16]. Five-day old calli were irradiated in a semi-automated fashion, induced for regeneration and then selected on bialaphos containing medium. Out of 600 treated immature embryos, two plants could be recovered, which were resistant to the herbicide and showed integration of the transgene [16]. No reduction of the regeneration capacity was found for laser treated calli compared to untreated calli, but noteworthy, laser treated calli (without transgene) produced significantly higher amounts of shoots per callus, which might be interpreted as a sort of laser induced proliferation [16].

Historically, the use of nanosecond laser pulses is the oldest approach for photoinjection and has been successfully used in various settings. However, only low injection rates have been published and the approach has now been almost completely replaced by NIR fs laser systems (see next section), which have a higher precision and significantly lower damage potential.

### Photoinjection in single plant cells with NIR fs laser pulses

As in photoinjection within the biomedical field, the rise of NIR fs laser bearing their high precision and low off-target effects resulted in a number of recent publications exploring this laser type for photoinjection of plant cells. Noteworthy, the first report of NIR fs laser photoinjection in plant cells was published even before the first report of NIR fs laser transfection in mammalian cells, which was the starting point of a large number of publications in this field [6, 11]. Single cells within *Arabidopsis thaliana* meristems were irradiated using a NIR fs oscillator (λ = 800 nm, ${t}_{P}$ = 180 fs, ${f}_{rep}$ = 80 MHz) to deliver propidium iodide to selected cells. Thus, intercellular communication based on dye diffusion could be determined in a cell type-dependent manner. This also demonstrated the intrinsic advantage of using this class of lasers: the near-infrared wavelength is only slightly absorbed in the tissue and due to the multiphoton absorption process, which is necessary for perforation (see “Basic physics of photoinjection” section), the effect is only limited to the focal volume. Using an analogous approach with a blue CW laser (λ = 488 nm), cells in the laser path above the focus were loaded as well, thus rendering a single cell analysis impossible [11].

A more detailed investigation of photoinjection was performed using a comparable NIR fs laser source. The applied energy density was deliberately chosen below the calculated threshold for optical breakdown, so that the authors assumed that the permeabilization of the membrane took place in the low-density plasma regime (see “Basic physics of photoinjection” section) [63]. In fact, this was the only publication in the field of plant sciences that explicitly claimed to work in this regime. Some of the subsequent publications, both in the plant sciences and in the biomedical sector, even explicitly refer to the generation of a cavitation bubble as an indicator of successful photoinjection [70, 77]. The theoretical plasma expansion in the axial direction was calculated to be approx. 260 nm. The z-position of the focus was varied in 0.5 µm steps over 3 µm to ensure that the membrane was hit by the small focus volume without irradiating areas multiple times. With optimized parameters, the authors achieved 68% delivery efficiency with fluorescently labeled dextrans as marker molecules. From the time course of the fluorescence increases, a pore opening time of approximately 100 s was derived with an estimated pore size of 2–2.5 µm [63]. Since this information was read from the fluorescence images, it can be taken as a rough estimate of the dimensions only. For comparison, more elaborative investigations on the pore size using the low-density plasma regime in mammalian cells calculated a pore diameter in the range of 80 nm and transmission electron microscopy images of laser irradiated and chemically fixed cells indicated a pore diameter in the range of 0.5 to 1.0 µm [58, 92].

**Fig. 3:**
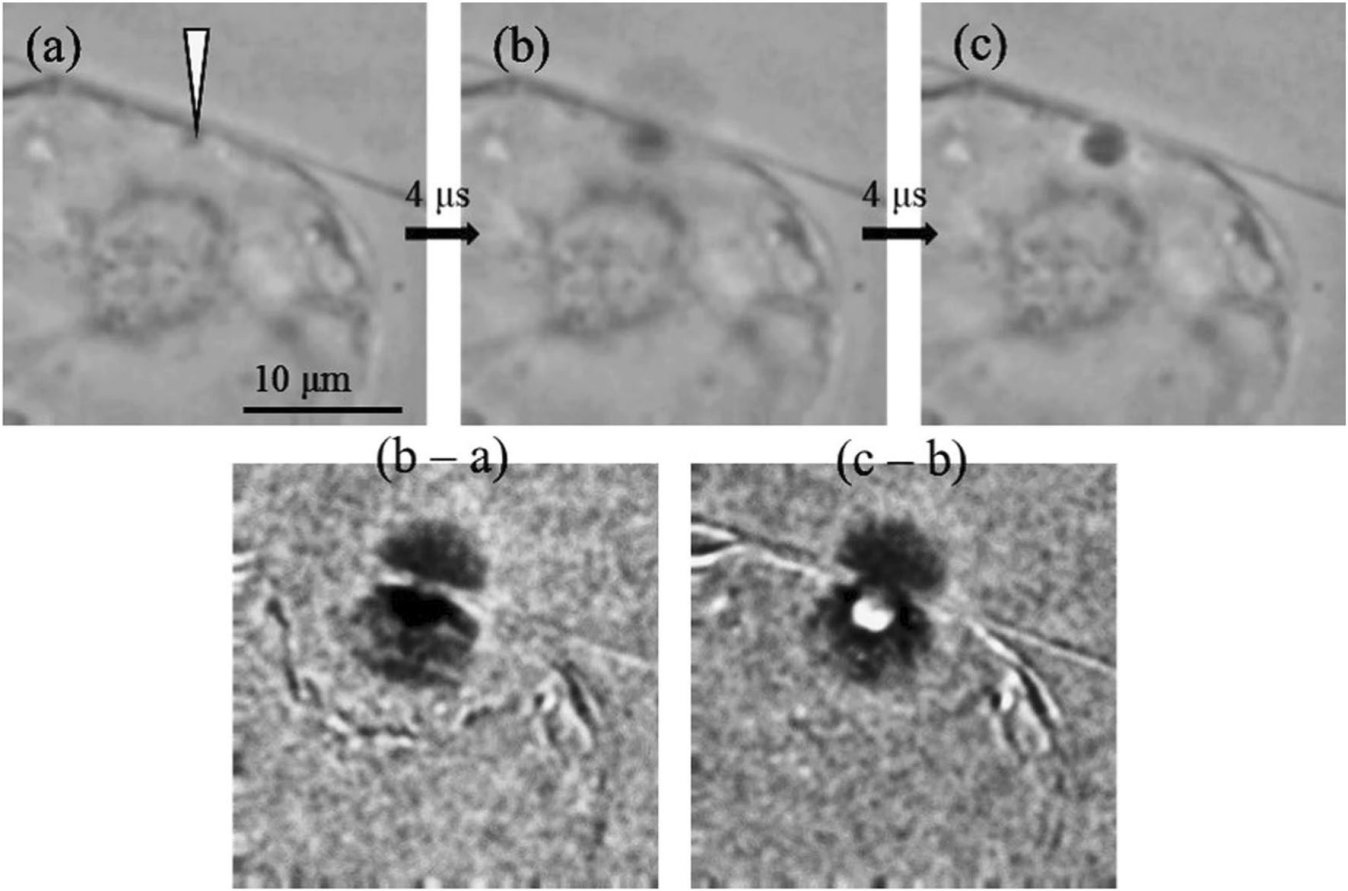
high-speed imaging of a photoinjection event in the photodisruptive regime. The focal point is depicted by a white arrow in (**a**). The generation and progression of a short lived cavitaion bubble can be observed 4 µs (**b**) and 8 µs (**c**) after application of a single laser pulse. The differential images shown in the bottom row reflect the dynamics of the photoinjection. (image reprinted from Rukmana et al. [68])

The positioning of the laser focus with sub-µm precision on the cell membrane or cell wall represents a major challenge in photoinjection [70, 93]. To account for this, different focus geometries previously used in mammalian cells have been investigated: a single focus of a Gaussian beam, three axially offset foci, and a Bessel beam [19, 20, 22, 94]. The latter represents a special beam geometry, which is generated with the help of an axicon, a cone-shaped lens. This creates an elongated, needle-shaped focus with self-healing properties, meaning that the beam reforms upon encountering an obstruction in its path [94]. This way the axial distance for successful photoinjection can be increased by a factor of approx. 20 (in this particular setup 26 µm ± 2 µm), thereby reducing the requirements on the focusing precision, while keeping the lateral resolution comparable to a conventional gaussian focus [93, 94]. All variants were compared based on the transport of propidium iodide as a marker molecule in tobacco BY2 cells. The triple focus and the Bessel beam gave comparable results. Although the efficiencies obtained with the triple focus were slightly higher (61%), this was associated with reduced cell viability compared to the Bessel beam [70, 93]. These studies show that optical design also has a massive impact on the practical feasibility of biological studies and therefore interdisciplinary collaboration can be very beneficial to the chances of success. In addition, the authors investigated the relationship between the osmolarity of the buffers used and the uptake efficiency. It was shown that under hypoosmolar conditions, ejection of cytoplasm occurred and, accordingly, no introduction of molecules was possible. With increasing osmolarity, this effect disappeared and the cells began to take up molecules [70]. This observation agrees with the previously described data for UV microbeam photoinjection [69, 87]. Since the molecular weight of the dextran molecules scales with their Stokes radius, it is not surprising that the number of molecules taken up decreases with increasing molecular size [70]. For intact cells (i.e. with intact cell wall), uptake of molecules with 70 kDa (or, respectively, a Stokes radius of 5.71 nm) could no longer be observed, which might correspond to the SEL of the cell wall [65–68]. Indeed, at least low uptake rates were observed in protoplasts of the same cell type in the same experiment [70]. Interestingly, uptake of significantly larger molecules has been described by other authors, including the studies employing fluorescently labeled or marker gene encoding DNA described before. One possible explanation for this lies in the combination of the SEL of the cell wall and the retention force achieved by hyperosmolar plasmolysis. The latter causes a rebound of the cell volume as soon as the cell membrane is punctured by a laser, leading to an inward volume flow that restores the original cell volume. The exchanged volume can be up to 10–20% of the cell volume [8, 69]. However, in this case the cell wall will be the size-limiting factor, so that only molecules that can diffuse freely through the cell wall will be taken up into the cell (see “General considerations on the physical transport of molecules into plant cells” section). If the cell wall is removed enzymatically and protoplasts are produced, this barrier is removed, but at the same time the volume expansion up to the rigid cell wall and thus the restoring force is missing. The reduced volume exchange could thus explain the lower uptake during photoinjection of protoplasts. The early publications using UV microbeams most likely employed sufficient energy to not only perforate the membrane but also the well wall when directed at a contact point between cell membrane and wall (or applied two consecutive pulses to achieve cell wall perforation) [8, 16, 69]. In this regard, the increased precision and reduced energy deposition of femtosecond lasers might actually be cumbersome. A series of recent studies does support this hypothesis and gives a possible solution for the NIR fs laser approach [68, 83, 84]. The authors use an incomplete digestion of the cell wall to increase its permeability to macromolecules while maintaining the stabilizing effect of the cell wall. Using a combination of partial enzymatic digestion, plasmolysis in hypertonic buffer and fs NIR laser irradiation (single laser pulse, λ = 800 nm, ${t}_{P}$ = 150 fs) of the cell membrane, the authors were able to transport FITC conjugated dextrans up to 2 MDa as well as polymer nanoparticles with a diameter of about 80 nm very efficiently into BY2 cells (Fig. 3) [68, 83, 84]. The success of the treatment was directly dependent on the pretreatment with enzyme solution and hypertonic buffer. Without digestion, only smaller dextrans (20 kDa) were able to diffuse through the cell wall and efficiently entered the cell upon photoinjection, whereas the 2 MDa dextrans were excluded. Upon enzymatic pre-treatment, both dextran sizes readily diffused through the cell wall and could be loaded into the cell [68, 83]. This approach was finally used to study intra- and intercellular diffusion behavior of nanoparticles in BY2 cells [84]. The authors further investigated the size cut-off for delivery by employing different sizes of particles. Whereas 80 nm styrene/boron-dipyrromethene (BODIPY) methacrylate particles and 2 MDa dextrans (Stokes radius approx. 27 nm) could be delivered across the cell membrane, 110 nm red fluorescent nanoparticles were excluded [84]. Although it is temping to define a maximum pore diameter and/or cut-off molecular weight using this kind of approach, it needs to be considered that various factors likely influence the uptake, including the charge, the three-dimensional structure as well as the flexibility of the applied nanoparticle or molecule.

As initially discussed (see “Historic view on laser-based molecular delivery” section), two basic regimes can be differentiated when applying NIR fs lasers for photoinjection: the low-density plasma regime which is based on photochemical effects and optical breakdown which results in the formation of cavitation bubbles with associated mechanical impact. The former is widely considered to be more precise and less toxic to cells. On the other hand, optical breakdown intuitively should result in larger membrane openings and accordingly to more efficient loading of large molecules, which is also supported by respective findings in mammalian cells [58]. Ultimately, this raises the question of the optimal balance between cell viability and photoinjection efficiency. The more recent studies imply that plant cells (at least in the case of BY2 cells and microalgae) are relatively robust and can therefore be treated efficiently by photoinjection in the optical breakdown regime [68, 70, 83, 84]. Indeed, the occurrence of a bubble as a prerequisite for successful photoinjection was described and the mechanical impact of the cavitation bubble was shown to support molecular delivery even if the focus was not directly aligned to the cell membrane [70, 84]. Another putative advantage is the possibility to treat cells with a single pulse from an amplifier system supporting the throughput, whereas the exposure times of oscillator systems are often given in the range of some 10 to 100 ms to achieve a sufficient effect. However, studies on this are limited and not available for plant cells, so far.

In sum, the application of NIR fs laser pulses (in the optical breakdown regime) represents the current gold standard for photoinjection. Still, only very limited data on its application in plant science is available.

### Alternative approaches for photoinjection in plant cells

Besides the classical regimes, some other approaches for photoinjection in plant science have been explored. For example, DNA-coated gold-micro-particles from a particle bombardment setup for plasmid delivery were applied instead of soluble plasmid DNA. The particles were placed directly on top of the target cells and then accelerated across the cellular membrane by a single pulse of an ArF laser (λ = 193 nm, ${t}_{P}$ = 10 ns). In primary explants of *Torenia hybrida* cv. Summerwave Blue, transient expression was observed in about 10% of the treated cells [95]. In another experiment, the particles could even be targeted to chloroplasts of *Nicotiana tabacum* cv. Xanthi guard cells, when the laser was applied directly at the cell surface on top of a chloroplast [96]. Besides being an interesting concept, the approach can rather be considered as a laser assisted particle bombardment method on a micrometer scale, than a photoinjection procedure.

In another interesting application, fs photoinjection of microalgae was demonstrated [82]. In this case, peptide aptamers were introduced into *Euglena gracilis* cells to determine intracellular paramylon concentration. The laser focus was automatically scanned over sedimented cells and the intracellular fluorescence of the introduced aptamers was correlated with the paramylon concentration. The authors also demonstrated the local selectivity of the laser treatment by spatially patterning of algae monolayers [82]. Interestingly, the approach was effective even though no specific focusing onto the microalgae was conducted. Although these results should be confirmed by further experiments, this represents an interesting perspective on the versatility of the methodic approach showing the applicability of photoinjection in very specific settings.

## Conclusion and future perspectives of photoinjection in plant sciences

The existing literature demonstrates the exciting potential of photoinjection methods in plant sciences to transport extracellular molecules into the cytoplasm — and in some specific applications even into organelles — of walled plant cells and protoplasts. When optimized parameters are applied, the approach proves to be very efficient and of low toxicity for the target cells. Whether its core feature, single cell selectivity, is advantageous or disadvantageous depends on the application. However, the potentially high efficiency in combination with modern automated approaches to sample processing, such as microfluidic focusing, could realistically achieve medium cell throughputs (hundreds to few thousands of cells per minute), which could yield relevant cell numbers for a variety of studies. Comparable approaches for automated throughput have been reported for mammalian cells in the biomedical field [19–23]. Considering the practicability and affordability of the approach, more cost-efficient laser sources should be explored in the future. Although NIR fs lasers theoretically provide the highest precision whereas UV (or visible) wavelength could induce off-target effects, there is no clear experimental evidence that this has a major impact in practice in the context of photoinjection. In contrast, only very limited data is available on picosecond laser systems for photoinjection, which could provide a good balance between precision and affordability. More fundamental research on the physics of photoinjection in plant cell is needed to allow relevant cost–benefit-estimations. In addition, there is a lack of literature for plant research on the higher throughput techniques already established in mammalian systems.

The available studies in plant science, in particular the more recent ones, demonstrate the technical feasibility of the methodology, but hardly show application beyond the basic proof-of-concept. There is a strong need for research in this area, where the photoinjection is not only considered as an isolated research field, but is used rather in the context of more complex biological questions. While historically the first publications focused on the production of transgenic plants by integrating the introduced genetic material into plant genomes, modern molecular tools are further exciting fields of application for photoinjection. In particular, the CRISPR/Cas9 technology and related genome editing methods could benefit from photoinjection as a directed transfer method. Since engineered Cas9 complexes contain NLSs, the further transport into the nucleus following photoinjection would not be an additional hurdle, the methods could perfectly complement each other to produce genome-edited cells [97]. Several of the above cited publications demonstrated the feasibility to regenerate plants from photoinjected cells and photoinjected zebrafish embryos could develop into fully functional animals [15, 16, 40, 69, 91]. This shows the potential for photoinjection being used not only in basic research but also in plant production. As an exciting specific application, photoinjection can potentially be used for the manipulation of pollen and was described as a potential future technology in this field [98]. However, the authors could not find any reports on the use of photoinjection in the field of pollen treatment until now.

The described applications do require strong interdisciplinary cooperation at the interface of optical technologies and plant science and it was recognized as early as 1992 that the continued success of the methodology heavily relies on this intense collaboration [99]. The most recent publications demonstrate, that not only the physical interactions are relevant to successful photoinjection, but also the sample pretreatment and handling play a major role. With the present review, we hope to inform potential interested plant scientists about the possibilities as well as current limitations of the technique and thus give new impulses for the transfer of photoinjection into the field of plant science.

## Data Availability

Not applicable.

## Abbreviations

CW: Continuous wave
fs: Femtosecond
GUS: Glucuronidase
LDP: Low-density plasma
LISW: Laser induced shock waves
NA: Numerical aperture
NIR: Near infrared
NLS: Nuclear localization sequence
ROS: Reactive oxygen species
SEL: Size exclusion limit
UV: Ultra violet
VIS: Visible
