# Correction to: Laser‑based molecular delivery and its applications in plant science

**DOI:** 10.1186/s13007-022-00936-5

**Published:** 2022-08-25

**Authors:** Dag Heinemann, Miroslav Zabic, Mitsuhiro Terakawa, Jens Boch

**Affiliations:** 1 Hannover Centre for Optical Technologies, Leibniz University Hannover, Nienburger Str. 17, 30167 Hannover, Germany; 2 Institute of Horticultural Production Systems, Leibniz University Hannover, Herrenhäuser Str. 2, 30419 Hannover, Germany; 3 Cluster of Excellence PhoenixD, Leibniz University Hannover, Welfengarten 1, 30167 Hannover, Germany; 4 Department of Electronics and Electrical Engineering, Keio University, 3-14-1 Hiyoshi, Kohoku-ku, Yokohama, 223-8522 Japan; 5 Institute of Plant Genetics, Leibniz University Hannover, Herrenhäuser Str. 2, 30419 Hannover, Germany

## Correction to: Plant Methods (2022) 18:82 10.1186/s13007-022-00908-9

In the original version of the article the wrong figure appeared as Fig. 1; Fig. 1 should have appeared as shown in this correction.

**Fig. 1:**
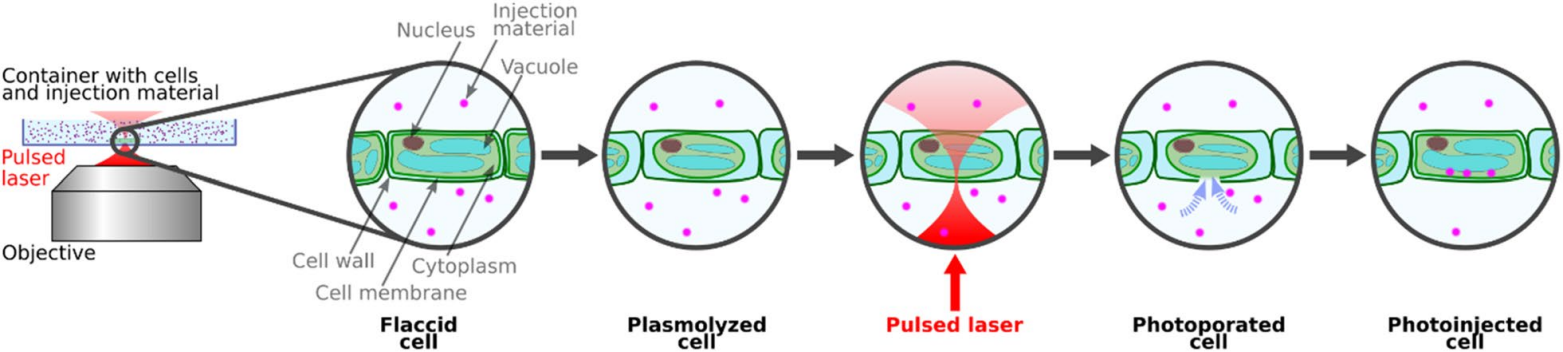
Sketch of a typical photoinjection experiment using an inverted microscopic setup and a pulsed laser source. The laser beam is focused onto the sample using an inverted microscope setup. A single laser pulse or a train of pulses facilitates of the cellular membrane and possibly the cell wall. The exact physical process of photoporation depends on the applied laser parameters and will be discussed in the following section. Plasmolyzing the plant cell prior to photoinjection supports the molecular uptake

The original article [1] has been corrected.
